# “TiC-TUG”: technology in clinical practice using the instrumented timed up and go test—a scoping review

**DOI:** 10.1007/s40520-024-02733-7

**Published:** 2024-04-27

**Authors:** Melissa J. Böttinger, Sarah Labudek, Daniel Schoene, Carl-Philipp Jansen, Marios-Evangelos Stefanakis, Elena Litz, Jürgen M. Bauer, Clemens Becker, Katharina Gordt-Oesterwind

**Affiliations:** 1 Digital Unit, Center for Geriatric Medicine, Heidelberg University Hospital, Heidelberg, Germany; 2 Network Aging Research, Heidelberg University, Bergheimer Str. 20, 69115 Heidelberg, Germany; 3 Clinic for Psychiatry and Psychotherapy, Helios Hospital Schwerin, Schwerin, Germany; 4 Institute of Radiology, University Hospital Erlangen, Erlangen, Germany; 5 Department of Clinical Gerontology and Geriatric Rehabilitation, Robert Bosch Hospital, Stuttgart, Germany; 6 Institute of Sports and Sports Sciences, Heidelberg University, Heidelberg, Germany

**Keywords:** Timed up and go test (TUG), Instrumented assessment, Technology-based assessment, Clinical outcome assessment, Physical capacity, Mobility, Older people, Aged [MeSH]

## Abstract

Digitized assessments have a considerable potential to guide clinicial decision making and monitor progress and disease trajectories. The Timed Up and Go test (TUG) has been long established for assessment in geriatric medicine and instrumented versions (iTUG) have been developed and validated. This scoping review includes studies that applied the iTUG and aims to identify use cases to show where and how iTUG assessment could guide interventions and clinical management. The literature search was limited to peer-reviewed studies that performed pre- and post-intervention measurements with a 3-meter TUG instrumented with body-worn technology in samples of at least 20 subjects aged 60+ years. Of 3018 identified articles 20 were included. Four clinical use cases were identified: stratification for subsequent therapy, monitoring of disease or treatment-associated changes and evaluation of interventions in patients with idiopathic normal pressure hydrocephalus (1), and patients with Parkinson’s disease (2); monitoring after joint replacement surgery (3), and evaluation after different exercise and rehabilitation interventions (4). The included studies show diversity in terms of iTUG technology and procedures. The identified use cases highlight clinical relevance and high potential for the clinical application of the iTUG. A consensual approach as well as comprehensive reporting would help to further exploit the potential of the iTUG to support clinical management. Future studies should investigate the benefits of segmental iTUG analysis, responsiveness and participants’ perspectives on clinically meaningful changes in iTUG.

**Supplementary Information:**

The online version contains supplementary material available at 10.1007/s40520-024-02733-7.

## Introduction

Mobility is a key indicator of quality of life in older people [1]. The measurement of physical activity and capacity as important determinants of mobility has made tremendous progress over the last decade. While physical activity measurements (‘what DO they do?’) focus on actual performance in real life (e.g., daily step count) [2], physical capacity tests (‘what CAN they do?’) assess the ability to perform physical tasks [3]. It has been demonstrated that capacity testing can be used as a proxy of health status, to detect preclinical disability, to predict future outcomes, to identify groups of people at risk, and to monitor changes over time in individuals or groups of older people [3, 4]. Among the most commonly physical capacity assessments used in older populations are the Timed Up and Go test (TUG) [5], the Short Physical Performance Battery [6], Chair Rise maneuvers [7], the six-minute walk test [8] and gait speed measurements at habitual and fast pace.

During the TUG, the test person stands up from a chair, walks 3 m at usual pace, turns around, walks 3 m back, and sits down on the chair. The time needed to complete the TUG is recorded by a trained assessor. The TUG test procedure can be considered as special among the most established physical capacity tests for older people, as it combines different routine movements (e.g., sit-to-stand, gait initiation) and multiple directions of movement (e.g., forward walking, 180 degree turning). The TUG is popular for its simplicity [9], as it requires minimal equipment and space. Since more than 30 years the TUG has been established as a clinical standard test to study mobility during the aging process, also in the context of many age-associated diseases and geriatric syndromes [5]. Due to its profoundly established validity and reliability the World Health Organization recommends the TUG for balance assessment in older people in clinical and research settings [10].

More recently, instrumented versions of the TUG (iTUG) have been developed and validated in older populations with and without specific pathologies. For example, one study showed that the iTUG using portable inertial sensors was able to detect specific gait deficits in patients with Parkinson’s disease (PwPD) and that the results of iTUG components were associated with disease severity [11]. The authors of another recent study found that a smartphone-based iTUG applied in community-dwelling older people could predict scores of the Community Balance and Mobility Scale (CBM), a valid, reliable and comprehensive performance-based assessment for measuring physical function in older people [12].

Previous reviews on the iTUG focused on the different technologies utilized for TUG instrumentation [13], feasibility of the iTUG in mobile devices [14], and technological proposals in studies on fall risk analysis [15]. Two major approaches have matured to objectively and accurately capture a subject’s gait pattern: wearable inertial measurement units (IMUs) and non-portable systems such as camera-based optical motion capturing [13]. Unlike stopwatch measurement, instrumented approaches allow the segmentation of the TUG, i.e., sit-to-stand, walk 1 (away from chair), turn 1 (around marker), walk 2 (back to chair), turn 2 (before sitting), and stand-to-sit movement [13].

There is consensus that benefits of the iTUG compared to the traditional stopwatch version include additional data on performance parameters, objective measurement, automated data collection and processing that can facilitate long-term analysis. In addition, the iTUG has the potential to be self-administered in the home environment of older people [13–15]. However, there are certain barriers to the implementation of instrumented tests, such as cost for buying medically certified products, and additional training of assessment staff [13]. In addition, kinematic parameters, such as angular velocity, are still unfamiliar to healthcare professionals.

From the perspective of physiotherapists, geriatricians, other medical specialists, and allied health care professionals, there is a growing interest to understand how the iTUG could guide clinical management and interventions in older patients. The development and implementation of technologies as measurement tools in the clinical setting must also be compliant with the regulatory requirements for medical devices [16]. The Food and Drug Administration (FDA) and the National Institutes of Health (NIH) developed the “Biomarkers, EndpointS and other Tools” (BEST) recommendations [17] to implement, harmonise the terminology and advance the adoption in translational medicine and medical product development. It describes distinct use cases for digital biomarkers and clinical outcome assessments.

Our main research question was to identify studies that have used the total iTUG duration to examine differences before and after an intervention. Secondly, we were interested if the identified studies also used a segmental analysis of the TUG maneuver. Finally, we wanted to describe the applied technology and procedures (e.g., placement of the device, number of repetitions etc.). Our research interest and search strategy included the identification of other geriatric use cases for the iTUG to guide clinical decision making.

This scoping review is part of the SMART-AGE project (P2019-01-003 + https://smart-age.psychologie.uni-heidelberg.de/). Within this project, the iTUG is being used for process evaluation of the Keep On Keep Up App (KOKU). This app was developed by the University of Manchester [18]. Final results of the SMART-AGE project are expected in 2025.

## Methods

### Protocol and registration

This report adheres to the Preferred Reporting Items for Systematic Reviews and Meta-Analyses extension for Scoping Reviews (PRISMA-ScR) [19] (Supplement 1). The study protocol has been registered prior to the screening process (https://osf.io/exjz2/). A preliminary search in PubMed and PROSPERO was conducted and no current or ongoing reviews on the topic were identified.

### Eligibility criteria

We were interested in identifying studies with different populations of older adults, including those with common age-associated diseases, evaluating surgical, pharmaceutical, training and other types of interventions by means of the iTUG. Studies needed to apply an instrumented version of the TUG in a sample with a mean age of 60 years and above. Studies needed to use wearable technologies for the iTUG assessment as only the latter provide an increased clinical utility when compared to more complex systems, i.e., ambient or camera systems that cannot be used in outpatient settings. Peer-reviewed studies published since 2012 were included as the technologies developed prior to the last ten years might not meet the current technological standards. Studies needed to measure the effect of an intervention over at least two timepoints. Due to the limited power of smaller pilot studies, trials were required to include at least 20 individuals in their analysis.

Studies were excluded based on populations experiencing congenital, non-age-related, or orphan diseases. Studies using the iTUG at baseline to predict outcomes such as falls without a follow-up measurement were not included. The articles had to comply with the standard version of the TUG using the 3 m distance [5]. Many home-dwellers have spatial restrictions not allowing gait assessments of more than 3 m. A full list of the inclusion and exclusion criteria can be found in Supplement 2.

### Search and selection of sources of evidence

To identify potential articles, the electronic databases PubMed/MEDLINE, CINAHL, Web of Science, Cochrane Library and IEEE Xplore were searched on July 6, 2022. Reference lists of three relevant reviews on the iTUG [13–15] and of included articles were searched.

Search fields included title, abstracts, and Medical Subject Headings (MeSH). The search strategy used in PubMed/MEDLINE can be found in Supplement 3.

Titles and abstract screening was conducted by two reviewers (MJB, SL) independently using Rayyan (http://rayyan.qcri.org; [20]). Full-text screening was conducted independently by at least two reviewers (MJB, SL, KGO). Any disagreements were solved through discussion.

### Data extraction process

Two reviewers (MJB, SL) filled out the data charting form independently. A third reviewer (KGO) was involved in case of conflict.

The following article characteristics were extracted: (a) general characteristics of the included studies (first author, year of publication, country in which study was conducted, population, sample size, sex, age, recruitment setting, study design, intervention description), (b) iTUG-related study characteristics (iTUG distance, technology, type of sensors, speed instruction, body placement of the technology used, number of iTUG repetitions, analysed segments, purpose of instrumentation), (c) pre-post differences within groups reported for iTUG total duration (pre- and post-intervention mean total iTUG duration) and pre-post differences within groups reported for iTUG segments sit-to-stand, walk 1, turn 1, walk 2, turn 2, stand-to-sit (improvement/worsening compared to baseline). In case of missing information, the articles’ corresponding authors were contacted via email or ResearchGate up to two times in intervals of two weeks.

### Critical appraisal of individual sources of evidence

Methodological quality of the articles was assessed using the “before-after (pre-post) studies with no control group” Quality Assessment Tool of the United States National Institute of Health (https://www.nhlbi.nih.gov/health-topics/study-quality-assessment-tools). The items were specified according to the purpose of this scoping review, e.g., the item on assessment was explicitly rated regarding iTUG assessment. Quality assessment was conducted independently by at least two reviewers (MJB, SL, KGO). In case of disagreement, the results were discussed until consensus was reached. A detailed description of the applied decision criteria are provided in Supplement 4.

### Synthesis of results

Results are reported in clusters of diagnoses or interventions, mainly in form of tables. Along the screening process, we decided to split the reporting of results into two parts: first, we report the changes in total iTUG durations before and after intervention, followed by the changes in iTUG segments.

## Results

The search yielded 3018 records of which 312 underwent full-text review. Of these, N = 20 records were included. The PRISMA-ScR flow diagram [19] containing the number of identified and excluded articles at each stage of the search and selection process is displayed in Supplement 5.

The included studies were divided into five groups: Patients with idiopathic normal-pressure hydrocephalus (PwiNPH) [21–24], Patients with Parkinson’s disease (PwPD) [25, 26], elective orthopedic surgery [27, 28], exercise and rehabilitation interventions [29–38], and assistive devices [39, 40].

### Study characteristics

The average sample size of the included studies was 43 (range: 20–119) with five (25%) studies including more than 50 individuals (Table 1). The mean age of the samples ranged from 60 to 91 years. Most of the studies (n = 11) were conducted in inpatient settings, i.e., hospital, clinic, or inpatient rehabilitation. Other settings were community-dwelling (n = 4), outpatient rehabilitation/clinic (n = 2), residential care/care home (n = 2), and day care center (n = 1). Ten studies included physical exercise interventions (e.g., Tai Chi), two applied drug therapies (i.e., l-dopa), five examined effects of surgical or invasive procedures (e.g., arthroplasty, cerebrospinal fluid tap-test) and two studies applied assistive devices (i.e., vibratory stimulation, orthosis). Depending on the type and duration of the respective intervention, participants were measured on the same day (e.g., with and without orthosis), within a couple of days (e.g., 72 h after cerebrospinal fluid tap-test), or weeks after the end of treatment (e.g., 12 weeks after intervention).

**Table 1:** General characteristics of included studies (n = 20)

First author	Year	Country	Population, sample size, sex	Mean age ± SD	Recruitment setting	Study design	Intervention, description
Patients with idiopathic normal pressure hydrocephalus (PwiNPH)—surgical and invasive procedures							
Ferrari	2020	Italy	“pure” (p-iNPH): 45 (31 m/25f) “secondary” (s-NPH): 20 (12 m, 8f)	p-iNPH: 73.9 ± 5.0 s-iNPH: 76.7 ± 3.1	Hospital	Case–control study	Tap-test: removal of 30–40 ml of cerebrospinal fluid
Ferrari	2022	Italy	42 (15 m/27f) PwiNPH	75.2 ± 4.0	Hospital	Cohort study	Tap-test: removing 30 ml of cerebrospinal fluid Ventriculoperitoneal shunt (VPS) surgery
Ishikawa	2019	Japan	IG: 32 (22 m/10f) PwiNPH CG: 87 (40 m/47f) active controls	IG: 77.6 ± 5.5 CG: 79.4 ± 7.0	Hospital	Case–control study	IG: tap-test: removal of 30 ml of cerebrospinal fluid, VPS surgery CG: 3-h Activity- Keeping Program 1-2x/week, individual exercises, group exercises, and brain training
Yamada	2019	Japan	Tap-test-positive: 28 (18 m/10f) Tap-test-negative: 8 (2 m/6f) 18 (12 m/6f) VPS surgery (if tap-test-positive)	Tap-test-positive: 77.5 ± 5.9 Tap-test-negative: 78.3 ± 9.0 VPS surgery: 76.6 ± 5.1;	Hospital and rehabilitation facility	Cohort study	Tap-test, removal of 30–40 ml of cerebrospinal fluid VPS surgery
Patients with Parkinson’s disease (PwPD)—pharmacological intervention							
Dibilio	2017	Italy	28 PwPD	66.3 ± 8.5	Hospital^a^	Cohort study	L-dopa ON/OFF, measured during “OFF” state (before first daily L-dopa dose after overnight fast) and during “ON” state (peak of dose)
Miller Koop	2018	USA	30 (18 m/12f) PwPD	61.9 ± 9.0	Hospital	Cohort study	L-dopa Equivalent Daily Dose: 513.1 ± 256 mg, measured on 2 days (regular medication and off medication for > 12 h)
Orthopedic conditions—elective surgery							
Bloomfield	2019	Canada	68 (34 m/34f) patients with osteoarthritis of the knee	67.5 ± 9.8	Hospital	Cohort study	Primary unilateral total knee arthroplasty
Perelgut	2020	Canada	23 (14 m/9f) patients receiving a collared femoral stem 26 (15 m/11f) patients receiving a collarless femoral stem	Collared: 64.1 ± 8.2 Collarless: 65.0 ± 8.0	Hospital	Randomized controlled trial	Collared or collarless femoral stem received through a direct anterior approach for primary total hip arthroplasty
Exercise and rehabilitation interventions in different settings and populations							
Patients with Parkinson’s disease (PwPD)							
Flood	2020	Ireland	IG: 12 (9 m/3f) PwPD CG1: 8 (5 m/3f) PwPD CG2: 14 (9 m/5f) healthy controls	IG: 74.8 ± 5.9 CG1: 78.1 ± 4.9 CG2: 70.2 ± 5.9	Hospital	Case–control study	IG: 16 1-h face-to-face LSVT-BIG sessions, frequency/duration: 60 min each, 4 sessions per week for 4 weeks; CG1: no physical therapy CG2: same experimental protocol as IG
Mollinedo-Cardalda	2018	Spain	IG: 13 (5 m/8f) PwPD CG: 13 (4 m/9f) PwPD	IG:62.9 ± 9.8 CG: 66.0 ± 13.1	Community-dwelling	Randomized controlled trial	IG: Mat Pilates with TheraBand® and 0.5 kg ankle and/or wristbands, 7 exercises in 3 sets of 8 repetitions; 12 weeks, 2x/week for 60 min CG: calisthenics exercises; 12 weeks, 2x/week for 60 min
Picardi	2020	Italy	20 (15 m/5f) PwPD	74.9 ± 5.4	Inpatient rehabilitation	Cohort study	Physiotherapy and occupational therapy at one-on-one sessions, frequency/duration: 6 weeks with 55 ± 25 physiotherapy sessions and 16 ± 8 occupational therapy sessions
Participants recruited in outpatient settings							
Smith	2021	Ireland	54 (5 m/49f) socially active adults aged 50+ years	63.6 ± 6.1	Community-dwelling	Cohort study	“Better Bones” strength, balance, and education program, frequency/duration: 1x/week for 6 weeks in community gym and tailored home program
Celletti	2020	Italy	44 (10 m/34f) patients experiencing lower back pain	70 ± 14.0	Outpatient rehabilitation clinic	Cohort study	10 sessions of back school, frequency/duration: 3x/week for 60 min
Doheny	2013	Ireland	33 (14 m/19f) healthy, sedentary middle-aged adults	59.8 ± 2.7	Community-dwelling	Cohort study	stepping program, frequency/duration: 4 weeks, 3x/week, increasing duration (1. week: 12 min, 2. week: 18 min, 3. + 4. week: 27 min); 11 participants exercised at home, 22 in the workplace
Williams	2021	UK	IG: 33 (19 m/14f) CG: 34 (21 m/13f) Patients with mild to moderate dementia	IG:78.6 ± 8.4 CG: 78.3 ± 8.0	Community-dwelling	Randomized controlled trial	IG: usual care + Tai Chi comprising Tai Chi classes, frequency/duration: 20 weeks, 1x/week for 90 min CG: medications and support services, social groups, peer support, iTUG data were collected at home
Participants recruited in inpatient or institutionalized settings							
Caronni	2019	Italy	25 (13 m/12f) elderly with peripheral neuropathy of the lower limbs	76.5 ± 6.7	Inpatient rehabilitation clinic	Cohort study	5–6 weeks rehabilitation including physiotherapy and occupational therapy
Cancela Carral	2017	Spain	36 (7 m/29f) participants aged 80+ years	87.9 ± 4.7	Day care centre	Randomized controlled trial	A: aerobic exercise program B: muscle resistance program C: joint mobility program; frequency/duration: 3 months, 3x/week for 45 min
Cancela Carral	2019	Spain	IG: 11 CG: 13 Females aged 85+ years	90.6 ± 4.4	Care home	Randomized controlled trial	IG: strength training for lower limbs, frequency/duration: 12 weeks, 2x/week for 60 min CG: maintain daily routines, handcraft, reading, cognitive stimulation, no systematic physical activity
Assistive devices							
Toosizadeh	2020	USA	10 (4 m/6f) older people with “moderate” function 10 (3 m/7f) older people with “low” function	“Moderate” function: 72.9 ± 2.8 “Low” function: 83.6 ± 9.5	Care home	Cohort study	Mechanical vibration with eccentric rotating servomotor, test with no vibration was performed first, followed by 30 or 40 Hz (randomized), participants were measured on same day with 2 min rest period between trials
Yalla	2014	USA	30 (7 m/23f) older people aged 65+ years	73 ± 6.5	Outpatient clinic	Cohort study	Tailored ankle foot orthoses with custom-made footplate and arch support with flexibility for plantar/dorsi flexion, participants were measured same day (shoe alone and shoe + orthoses)

*IG* intervention group, *CG* control group, *PwiNPH* patients with idiopathic normal pressure hydrocephalus, *p-iNPH* pure idiopathic normal pressure hydrocephalus, *s-NPH* secondary normal pressure hydrocephalus, *PwPD* patients with Parkinson’s disease, *LSVT-BIG®* Lee Silverman Voice Treatment, *VPS *ventriculoperitoneal shunt

^a^information received on request from the authors of the respective article

### iTUG related characteristics

The instructions on walking speed ranged from comfortable/preferred/habitual speed to as fast as possible (Table 2). The number of repetitions per assessment varied from one repetition up to six, with most studies (n = 18) conducting two or more repetitions. The majority of the studies (n = 15) used stand-alone wearable inertial sensors, three studies used sensors embedded in mobile devices, i.e., an iPhone. Two studies used a combination of both, i.e., inertial sensors and iPod Touch. Most studies used accelerometers measuring acceleration (m/s^2^) and gyroscopes measuring angular velocities (deg/s) (n = 19). In eight studies, magnetometers measuring the direction of Earth’s magnetic field were used additionally. The sensors were attached most often to the lumbar spine (n = 14). Other locations were shin, thigh, shoe, or navel. Different methods were used for attachment (Table 2).

**Table 2:** iTUG-related study characteristics (N = 20)

First author (year)	iTUG technology, sensors	iTUG speed instruction	Body placement	Number of iTUG repetitions	Analyzed segments	Purpose of iTUG use
Patients with idiopathic normal pressure hydrocephalus (PwiNPH)—surgical and invasive procedures						
Ferrari (2020)	“mGAIT” (mHealth Technologies, Bologna, Italy): accelerometer, gyroscope	Preferred speed^a^	Shoes, lower trunk	3	Total iTUG duration	iTUG was used to determine contribution of instrumented measures in assessing motor performance pre vs. post tap-test in PwiNPH; evaluate differences in effects 24 h or 72 h after tap-test
Ferrari (2022)	“mGAIT” (mHealth Technologies, Bologna, Italy): accelerometer, gyroscope	Preferred speed^a^	Shoes, lower trunk	3	Total iTUG duration 3 segments: sit-to-stand, walk total, stand-to-sit	iTUG was used to test validity of instrumental variables: concurrent validity, responsiveness to VPS, ability in tap-test to predict VPS outcomes
Yamada (2019)	“iPhone” (Apple Inc., Cupertino, CA, USA), free application (Senior Quality, Digital Standard Co., Ltd., Osaka, Japan): accelerometer, gyroscope	As fast as possible	Pouch, navel	2	Total iTUG duration	iTUG was used to investigate different iTUG measures for evaluating gait disturbance pre/post tap-test and VPS
Ishikawa (2019)	“iPhone” (Apple Inc., Cupertino, CA, USA), free application (Senior Quality, Digital Standard Co., Ltd., Osaka, Japan): accelerometer, gyroscope	As fast as possible without running	Elastic belt, navel	2	Total iTUG duration 6 segments: sit-to-stand, walk 1, turn 1, walk 2, turn 2, stand-to-sit	iTUG was used to examine usefulness of iTUG in PwiNPH and to explore changes in gait patterns
Patients with Parkinson’s disease (PwPD)—pharmacological intervention						
Dibilio (2017)	“BTS G-WALK” (BTW Bioengineering S.p.A., Italy): accelerometer, magnetometer, gyroscope	Usual walk^a^	Waist belt, L4/L5	2	Total iTUG duration 6 segments: sit-to-stand, walk 1, turn 1, walk 2, turn 2, stand-to-sit	iTUG was used to evaluate L-dopa effects on gait parameters in PwPD disease in OFF and ON state
Miller Koop (2018)	“iPad” (Apple Inc., Cupertino, CA, USA), custom application (Cleveland-Clinic-Mobility and Balance application, CC-MB): accelerometer, gyroscope	Safe and comfortable speed^a^	Band, lumbar spine	2	total iTUG duration 4 segments: sit-to-stand, walk total, turn 1, turn to sit	iTUG was used to detect differences in performance of PwPD ON and OFF medication within the segments of iTUG
Orthopedic conditions—elective surgery						
Bloomfield (2019)	“iPod Touch” (Apple Inc., Cupertino, CA, USA), inertial sensors (MetaMotionR, MBientLab, San Francisco, CA, USA): accelerometer, magnetometer, gyroscope	Normal speed^a^	Above and below each knee	3	Total iTUG duration 5 segments: sit-to-stand, walk 1, turn 1, walk 2, stand-to-sit	iTUG was used to examine influence of functional parameters on postoperative outcomes after total knee replacement
Perelgut (2020)	Authors refer to Bloomfield (2019)				Total iTUG duration	iTUG was used to evaluate changes in clinical function after hip replacement surgery
Exercise and rehabilitation interventions in different settings and populations						
Patients with Parkinson’s disease (PwPD)						
Flood (2020)	“Trigno” (Delsys Inc., Natick, MA, USA): accelerometer	Comfortable and natural speed^a^	L5, above rectus femoris (thighs) and tibialis anterior (shins)	2	Total iTUG duration	iTUG was used to examine motor function of PwPD following LSVT-BIG® therapy
Mollinedo-Cardalda (2018)	“Wiva®” sensor: (LetSense SRL, Bologna, Italy): accelerometer, magnetometer, gyroscope	As fast as possible (no running)^a^	L4/L5	3 (1 practice trial fol-lowed by 2 repetitions of the TUG)^a^	Total iTUG duration 5 segments: sit-to-stand, walk 1, turn 1, walk 2, turn 2	iTUG was used to evaluate the effects of a mat Pilates program on dynamic balance in PwPD
Picardi (2020)	Intertial measurement unit (mHT-mHealth Technologies, Bologna, Italy): accelerometer, gyroscope	Preferred speed	Lower back	5	Total iTUG duration	iTUG was used to examine its validity as balance measure and responsiveness to rehabilitation in PwPD
Participants recruited in outpatient settings						
Smith (2021)	“Kinesis QTUGTM” instrument (Kinesis Health Technologies, Dublin, Ireland): accelerometer, gyroscope	As fast as possible	Elastic bandages, anterior tibia (shins)	1^a^	Total iTUG duration 5 segments: sit-to-stand, walk total, walk 1, turn 1, stand-to-sit	iTUG was used to examine its responsiveness, minimum detectable change (MDC) and observed effect size after a structured community exercise program
Celletti (2020)	“G-Sensor” (BTS SpA, Milan, Italy): accelerometer, magnetometer, gyroscope	Habitual speed^a^	Elastic belt, L5	3^a^	Total iTUG duration 5 segments: sit-to-stand, walk 1, turn 1, walk 2, stand-to-sit	iTUG was used to evaluate functional improvement after back school therapy
Doheny (2013)	Inertial sensors (Shimmer Research, Dublin, Ireland): accelerometer, gyroscope	As fast as possible^a^	L4: double-sided tape, tibial tuberosity: velcro straps	6 (1 practice trial followed by 6 repetitions of the TUG test with 1 min rest between repetitions)	Total iTUG duration 4 segments: sit-to-stand, walk to sit, turn 2, stand-to-sit	iTUG was used to evaluate functional mobility after step exercise program
Williams (2021)	Miniature balance sensor device (THETAmetrix, Portsmouth, UK): accelerometer, gyroscope	No specific instruction^a^	Elasticated strap, lower back	1	4 segments: sit-to-stand, walk 1, turn 1, walk 2	iTUG was used to evaluate the effectiveness of a Tai Chi intervention in people with dementia
Participants recruited in inpatient and institutionalized settings						
Caronni (2019)	Inertial measurement unit (mHT-mHealth Technologies, Bologna, Italy): accelerometer, gyroscope	Comfortable speed	Lower trunk	5	Total iTUG duration 5 segments: sit-to-stand, walk 1, turn 1, walk 2, turn and sit	iTUG was used to evaluate the responsiveness to rehabilitation of balance, gait and sensory ataxia measures in elderly with peripheral neuropathy of the lower limbs
Cancela Carral (2017)	“Wiva®” sensor: (LetSense SRL, Bologna, Italy): accelerometer, magnetometer, gyroscope	As fast as possible^a^	L4/L5	2 (with 3 min rest period)^a^	Total iTUG duration 5 segments: sit-to-stand, walk 1, turn 1, walk 2, stand-to-sit	iTUG was used to evaluate the effects of three chair-based exercise programs on strength, balance, functional mobility
Cancela Carral (2019)	“Wiva®” sensor: (LetSense SRL, Bologna, Italy): accelerometer, magnetometer, gyroscope	As fast as possible^a^	L4/L5	2 (with 3 min rest period)^a^	Total iTUG duration 5 segments: sit-to-stand, walk 1, turn 1, walk 2, stand-to-sit	iTUG was used to evaluate the effects of a strength training program on functional capacity
Assistive devices						
Toosizadeh (2020)	“LEGSys “ (BioSensics, Boston, MA, USA): gyroscope	Self-selected normal speed^a^	Anterior tibiae (shins)	3 (1 practice trial, 1 without vibration, 1 at 30 Hz, 1 at 40 Hz)	Total iTUG duration 5 segments: sit-to-stand, walk 1, turn 1, walk 2, turn and sit	iTUG was used to evaluate the effect of calf muscle vibration stimulation on dynamic balance
Yalla (2014)	“BalanSens™” (BioSensics LLC, Boston, USA): accelerometer, magnetometer, gyroscope	Habitual speed^a^	Lower back: waist belt, anterior tibiae (shins), thighs: velcro bands	2 trials for each footwear condition^a^	Total iTUG duration	iTUG was used to evaluate the effect of ankle foot orthoses on postural stability

Column 7 contains the analyzed variables/segments for which data regarding time measurements are available (see Tables 3 and 4)

*LSVT-BIG®* Lee Silverman Voice Treatment, *VPS* ventriculoperitoneal shunt

^a^Information received on request from the authors of the respective article

### Pre-post intervention differences of total iTUG duration and segmentation

Pre-post changes of the total iTUG duration as well as of the different iTUG segments are shown in Tables 3 and 4, respectively. Data on further kinematic iTUG measures such as angular velocity and acceleration were reported in 13 studies [21, 22, 25, 26, 29–36, 38].

**Table 3:** Pre-post differences within groups reported for iTUG total duration

Author (Year)	Compared groups/conditions	Pre-intervention: mean total iTUG duration (sec; ± SD)	Post-intervention: mean total iTUG duration post (sec; ± SD)					
Patients with idiopathic normal pressure hydrocephalus (PwiNPH) – surgical and invasive procedures								
Ferrari (2020)^a,c^		Before tap-test	24 h after tap-test	72 h after tap-test				
	Tap-test “pure” (p-iNPH) (n = 45)	17 (median)	16 (median)	15 (median)				
	Tap-test “secondary” (s-NPH) (n = 20)	22 (median)	20 (median)	18.5 (median)				
Ferrari (2022)^c^	Tap-test and ventriculoperitoneal shunt surgery (n = 42)	Before tap-test 24.1 ± 15.7		72 h after tap-test 19.5 ± 8.1				6 months after shunt 16.4 ± 5.5**
Yamada (2019)^a^		Before tap-test	24 h after tap-test	96 h after tap-test				
	Tap-test positive (n = 28)	23.8 ± 15.8	18.3 ± 10.2	15.1 ± 7.7				
	Tap-test negative (n = 8)	16.4 ± 6.8	18.0 ± 10.0	16.4 ± 7.6				
	Ventriculoperitoneal shunt surgery (n = 18, of those who were diagnosed tap-test-positive)	17.4 ± 5.0						After shunt 11.8 ± 3.1
Ishikawa (2019)		Before tap-test	N/A (no data for iTUG total duration changes available, data for single iTUG segments: see Table 4)					
	IG: tap-test/ventriculoperitoneal shunt surgery (n = 32)	19.4 ± 9.4						
	CG: activity program (n = 87)	12.5 ± 4.1						
Patients with Parkinson’s disease (PwPD)—pharmacological intervention								
Dibilio (2017)	Medication off vs. on (n = 28)	Off: 20.2 ± 12.6	On: 15.4 ± 5.2*					
Miller Koop (2018)	Medication off vs. on (n = 20)	Off: 8.2 ± 2.3 (median ± interquartile Range)	On: 7.4 ± 1.9* (median ± interquartile range)					
Orthopedic conditions—elective surgery								
Bloomfield (2019)		Before surgery	Baseline vs. 2w after surgery	Baseline vs. 6w after surgery		Baseline vs. 12w after surgery		
	Total knee replacement, moderate function (n = 46)	12.6 ± 2.4	21.8^b,^**	13^b^		11.7^b^		
	Total knee replacement, low function (n = 22)	21.6 ± 5.7	29.2^b^	18.7^b^		16.7^b,^**		
Perelgut (2020)		Before surgery	2w after surgery	52w after surgery				
	Total hip replacement, collared stem (n = 23)	13.0 ± 4.2^c^	16.3^b^	11.2^b,^**				
	Total hip replacement, collarless stem (n = 26)	13.4 ± 3.1^c^	15.6^b^	10.9^b,^**				
Exercise and rehabilitation interventions in different settings and populations								
Patients with Parkinson’s disease (PwPD)								
Flood (2020)^c^		Before intervention	2w after intervention	8w after intervention			13w after intervention	
	IG: PwPD, LSVT-BIG® therapy (n = 12)	9.4	7.7	7.7			7.5**	
	CG1: PwPD, no therapy (n = 8)	11.9	N/A (CG1 and CG2 were only measured at baseline)					
	CG2: healthy controls (n = 14)	8.5						
Mollinedo-Cardalda (2018)		Before intervention	End of intervention		4 weeks after intervention			
	IG: PwPD, mat pilates program (n = 12)	9.4 ± 2.4	7.8 ± 2.8**		7.8 ± 5.6			
	CG: PwPD, calisthenics exercises (n = 10)	9.1 ± 2.7	9.2 ± 2.5		9.6 ± 1.4			
Picardi (2020)	PwPD, inpatient rehabilitation (physiotherapy, occupational therapy) (n = 20)	Beginning of inpatient stay 15.8 ± 4.3	End of inpatient stay 14.1 ± 4.2*					
Participants recruited in outpatient settings								
Smith (2021)	Older people, “Better Bones” exercise program (n = 54)	Before intervention 6.3 ± 1.4	After intervention 5.8 ± 1.1**					
Celletti (2020)	Low back pain, back school (n = 44)	Before intervention 13.4 ± 3.9	After intervention 11.3 ± 2.2**					
Doheny (2013)	Healthy sedentary, step exercise (n = 33)	9.2 ± 1.2 (T1 = baseline); 8.8 ± 11.1 (T2 = 4w after T1, before start of intervention)	After intervention (T3) 8.9 ± 1.3					
Williams (2021)	IG: dementia, Tai Chi (n = 33)	N/A (no data for iTUG total duration changes available, data for single iTUG segments available, see Table 4)						
	CG: dementia, usual care (n = 34)							
Participants recruited in inpatient and institutionalized settings								
Caronni (2019)	Peripheral neuropathy lower limbs, inpatient physiotherapy/occupational therapy rehabilitation (n = 25)	Before rehabilitation 18.7 ± 7.8	End of rehabilitation 15.1 ± 5.2**					
Cancela Carral (2017)	80 + years	Before intervention	1w after intervention					
	Aerobic exercise (n = 13)	30.3 ± 21.4	26.6 ± 20.3					
	Muscle resistance exercise (n = 12)	29.1 ± 5.4	24.2 ± 8.4					
	Joint mobility exercise (n = 11)	30.3 ± 11.9	33.2 ± 13.6					
Cancela Carral (2019)	85 + years	Before intervention	Last week of intervention					
	Strength program (n = 11)	28.1 ± 10.0	27.7 ± 11.6					
	Control group (n = 13)	35.1 ± 11.6	38.6 ± 15.4*					
Assistive devices								
Toosizadeh (2020)^a^	Older people	No calf vibration	30 Hz		40 Hz			
	Vibratory stimulation, moderate function (n = 10)	11.3 ± 2.8	12.5^b^		12.9^b^			
	Vibratory stimulation, low function (n = 10):	24.0 ± 10.0	23.3^b^		23.5^b^			
Yalla (2014)	Older people, ankle foot orthosis (n = 30)	Shoe alone 13.8 ± 0.6	Shoe + ankle foot orthosis 14.1 ± 0.7					

*IG* intervention group, *N/A* data not available, *p-iNPH* idiopathic normal pressure hydrocephalus with pure hydrocephalus, *s-NPH* secondary normal pressure hydrocephalus, *LSVT-BIG®* Lee Silverman Voice Treatment, *w* weeks

^*^p < 0.05, ** p < 0.01 compared to baseline

^a^significance of pre-post differences not reported

^b^Calculated by the authors of this review

^c^Information received on request from the authors of the respective article, CG = control group

**Table 4:** Pre-Post differences within groups reported for iTUG total duration and segments

Author (year)	Groups	Pre-post conditions/timepoints	iTUG total	Sit-to-stand	Walk 1	Turn 1	Walk 2	Turn 2	Stand-to-sit	Notes
Patients with idiopathic normal pressure hydrocephalus (PwiNPH)—surgical and invasive procedures										
Ferrari (2022)	IG	Before tap-test vs. 6 months after ventriculoperitoneal shunt surgery (VPS)	+**	+**	Total walk:+**	N/A			+**	
Ishikawa (2019)	IG	Baseline vs. day after tap-test	N/A							CG: post-intervention data N/A
		Baseline vs. 1w after VPS			+*		+*	+*	+*	
		Tap-test vs. VPS			+*					
Patients with Parkinson’s disease (PwPD)—pharmacological intervention										
Dibilio (2017)	IG	Off vs. on medication	+*		+*	+**	+*	+**		
Miller Koop (2018)	IG	Off vs. on medication	+*			+*	N/A			
Orthopedic conditions—elective surgery										
Bloomfield (2019)	Moderate function	Baseline vs. 2w after TKR	−**					N/A		Data was extracted from bar chart, values and significances not reported for iTUG segments
		Baseline vs. 6w after TKR								
		Baseline vs. 12w after TKR								
	Low function	Baseline vs. 2w after TKR								
		Baseline vs. 6w after TKR								
		Baseline vs. 12w after TKR	+**							
Exercise and rehabilitation interventions in different settings and populations										
Patients with Parkinson’s disease (PwPD)										
Mollinedo Cardalda (2018)	IG (mat pilates)	Baseline vs. end of intervention	+**		+*		+*	+*	N/A	
		End of intervention vs. 4w after		−*			−*	+*		
	CG (calisthenics)	Baseline vs. end of intervention								
		End of intervention vs. 4w after						−*		
Participants recruited in outpatient settings										
Smith (2021)	IG (“Better Bones “)	Baseline vs. after intervention	+**	+**	+**	+*	N/A			
					total walk: +*					
Celletti (2020)	IG	Baseline vs. after back school treatment	+**	+*	N/A					Measurement timepoint not specified
Doheny (2013)	IG (step exercise)	Baseline (T1) vs. 4w after T1 (T2)			N/A			+*		T1 = first baseline, T2 = 4w after baseline before start of intervention
		Baseline (T1) vs. 4w after end of interv. (T3)			N/A			+*		
Williams (2021)	IG (Tai Chi)	Baseline vs. 6 months after baseline	N/A					N/A	N/A	
	CG (usual care)									
Participants recruited in inpatient and institutionalized settings										
Caronni (2019)	IG	Baseline vs. end of physiotherapy/occupational therapy rehabilitation program	+**	+**	+**	+**	+**	turn and sit: +*		
Cancela Carral (2017)	IG_A_ (aerobic)	Baseline vs. 1w after end of intervention			+*		+*	N/A		
	IG_B_ (resistance)			+*	+*		+*			
	IG_C_ (mobility)			−*		−*			−*	
Cancela Carral (2019)	IG (strength training)	Baseline vs. last week of intervention				−*		N/A		
	CG		−*	−*					−*	
Assistive devices										
Toosizadeh (2020)	Moderate function	No calf vibration vs. 30 Hz								Significance not reported for: iTUG total, sit-to-stand, walk1, walk 2
		No calf vibration vs. 40 Hz								
	Low function	No calf vibration vs. 30 Hz						+*		
		No calf vibration vs. 40 Hz				+*				

Articles that do not provide data on iTUG segments are not displayed in this table, + improvement, − worsening, *p < 0.05, **p < 0.01 compared to baseline

*CG* control group, *IG* intervention group, *N/A* not available, *TKR* total knee replacement, *VPS* ventriculoperitoneal shunt surgery, *w* weeks, *empty cells* pre-post difference is not statistically significant (trends for improvement/worsening are shown in Supplement 6)

The included studies showed a large variety for baseline iTUG results ranging from 6.3 to 35.1 s. A commonly used threshold for the total TUG performance to indicate normal vs. below normal mobility was 12 s [41]. In summary, eleven studies (55%) found a statistically significant reduction of the total iTUG duration after the interventions. Follow-up data were not available for two studies [24, 35]. The total iTUG duration at baseline (iTUG_B_, Table 3) and statistically significant pre-post intervention changes of the total iTUG durations as well as for segment durations are shown below for each group of participants studied. Supplement 6 provides information on trends (non-significant changes).

### Patients with idiopathic normal-pressure hydrocephalus (PwiNPH)

In the study of Ferrari et al. (2020) [21], participants with pure iNPH (p-iNPH, iTUG_B_ = 17.0 s) and secondary NPH (s-NPH, iTUG_B_ = 22.0 s) showed a shortening of the iTUG total duration 24 h after the lumbar tap-test. The changes were more pronounced after 72 h after the tap-test. It was however not reported whether these differences were statistically significant [21].

The other three studies [22–24] also applied the iTUG after a tap-test. Participants with a positive tap-test result (i.e., improvement of symptoms defined by ≥ 1 point on the iNPH grading scale [24]), subsequently underwent ventriculoperitoneal shunt surgery (VPS). Total iTUG duration at baseline was between 12.5 and 24.1 s [22–24]. A reduction of the iTUG total duration was observed in all three studies 24, 72 or 96 h after the tap-test. However, none reported interference-statistical values. A statistically significant reduction of the total iTUG duration was described by Ferrari et al. (2022) 6 months after shunt surgery [22].

One week after the VPS surgery significantly shorter durations were found by Ishikawa et al. [24] for both walking segments as well as turn2 and stand-to-sit compared to baseline. When comparing post-tap-test and post-shunt measurements the duration was significantly shorter for walk1 [24]. Patients in the study conducted by Ferrari et al. [22] showed a significant shortening for the sit-to-stand, total walk, and stand-to-sit segment duration 6 months after surgery.

### Patients with Parkinson’s disease (PwPD)

Two studies compared ON and OFF states of L-dopa medication in PwPD with a baseline total iTUG duration of 20.2 s in the study by Dibilio et al. [25] and 8.2 s in the study by Miller Koop et al. [26] at OFF state. Both studies observed significantly shorter total iTUG completion times in the ON versus OFF state. The time needed for turn1 was significantly shorter in both studies. In the study conducted by Dibilio et al. [25], both walking segments as well as turn2 were also significantly shorter during the ON state.

### Orthopedic conditions

Patients planned for elective total knee replacement were categorized as moderate (TUG_B_ = 12.6 s) and low function (TUG_B_ = 21.6 s) based on their total iTUG time before surgery in the study by Bloomfield et al. [27]. Only the moderate function group showed a statistically significant shorter total iTUG duration 2 weeks after surgery. The low function group showed a statistically significant shorter total iTUG time after 12 weeks.

Perelgut et al. [28] observed patients following total hip replacement using a collared (iTUG_B_ = 13.0 s) or collarless (iTUG_B_ = 13.4 s) femoral stem. Patients showed significantly shorter total iTUG durations 52 weeks after surgery in both groups [28].

### Exercise and rehabilitation interventions in different settings and populations

Three studies examined the effect of different exercise interventions in PwPD. Flood et al. [29] measured one PwPD intervention group participating in Lee Silverman Voice Treatment (LSVT-BIG®) therapy and two control groups (healthy control, no therapy control group). Mollinedo-Cardalda et al. [30] compared Mat Pilates with calisthenics. Picardi et al. [31] examined the effects of inpatient rehabilitation consisting of physiotherapy and occupational therapy [31]. The groups observed in the first two studies [29, 30] had a baseline total iTUG duration < 12 s and participants in the study of Picardi et al. [31] had a baseline value > 12 s (iTUG_B_ = 15.8 s). All studies found significantly shorter total iTUG durations in the PwPD intervention group following the intervention with persisting effects after 13 weeks in one study [29]. Three measures were shown to be responsive to rehabilitation (small to medium effect size): iTUG total duration, trunk angular velocity in the sit-to-stand and turning phases [31]. PwPD in the Mat Pilates group [30] showed significantly shorter times for both walking and turn2 segments after intervention. Four weeks after the end of the intervention, the significantly shorter duration for turn2 was maintained and a significantly longer duration was observed regarding sit-to-stand and walk2 [30].

Four studies examined pre-post iTUG results in outpatient settings. Smith et al. [32] measured participants of the 6-week “Better Bones” strength and balance exercise program (iTUG_B_ = 6.3 s) who had significantly shorter total iTUG durations after the intervention [32].

A significant decrease was observed for the sit-to-stand, walk1, turn1 and total walk segment duration [32]. Celletti et al. [33] used the iTUG to test the efficacy of an intervention in patients with low back pain (iTUG_B_ = 13.4 s). After ten sessions of the “Back School Therapy” intervention a significantly shorter time for the iTUG total performance was documented. The results also showed a significantly decreased sit-to-stand segment duration [33]. In the study from Doheny et al. [34] participants of a 4-week step exercise program (iTUG_B_ = 9.2 s) showed significantly shorter durations for the turn2 segment, however not for the total iTUG duration or other segments [34]. The controlled study from Williams et al. [35] investigated effects of a Tai Chi intervention in comparison to a non-exercising group of people with mild to moderate dementia. Information on total iTUG duration changes was not provided.

Three studies were realized in inpatient and institution settings. Caronni et al. [36] examined patients with peripheral neuropathy of the lower limbs (iTUG_B_ = 18.7 s). Study participants received 5 to 6 weeks of physiotherapy and occupational therapy. They showed a significantly shortened iTUG total time as well as decreased segment durations after the intervention [36]. Cancela Carral et al. (2017) [37] investigated people aged > 80 years participating in a 3-month study comparing aerobic (iTUG_B_ = 30.3 s), muscle resistance (iTUG_B_ = 29.1 s) or joint mobility (iTUG_B_ = 30.3 s) exercise programs. None of the groups showed significant pre-post differences for the total iTUG duration [37]. However, participants in the aerobic and muscle resistance exercise groups showed significantly shorter times for both walking segments. A significant decrease in sit-to-stand duration was observed in the muscle resistance exercise group, while the joint mobility exercise group showed significantly longer sit-to-stand, turn1 and stand-to-sit durations [37]. Nonagenarians participating in the study from Cancela Carral et al. [38] who took part in a 12-week strength training program (iTUG_B_ = 28.1 s) showed a a trend towards a reduction of total iTUG duration during the last week of the intervention [38]. Significantly longer total iTUG times were observed in the non-exercising control group (iTUG_B_ = 35.1 s). The results showed a significantly longer turn1 duration as well as a trend towards a reduction of walk1 and walk2 times in the intervention group, although theses results were not significant (see Supplement 6). In the control group, participants showed significantly increased sit-to-stand and stand-to-sit durations [38].

### Assistive devices

After applying calf vibration in two groups with different baseline levels for the iTUG (moderate function: iTUG_B_ = 11.3 s; low function: iTUG_B_ = 24.0 s), the results of the study from Toosizadeh et al. [40] showed trends towards longer iTUG total duration in the moderate function group and shorter iTUG total duration in the low function group [40] (see Supplement 6). Results on statistical significance were not reported for total iTUG duration. A significant decrease in duration regarding turn1 and turn and sit was observed in the low function group [40]. Participants of the study from Yalla et al. [39] wearing an ankle foot orthosis in combination with shoes (iTUG_B_ = 13.8 s) did not show significant changes regarding the total iTUG duration compared to wearing shoes alone.

### iTUG measurement properties reported in the included studies

Five studies reported good or excellent test–retest reliability of the iTUG [22–24, 26, 34]. One study [26] conducted a correlation analysis between the iTUG and the Movement Disorders Unified Parkinson’s Disease Rating Scale motor score (MDS-UPDRS-III), but it did not document a statistically significant correlation. Two studies [24, 32] found a strong correlation between manually measured TUG and iTUG durations. Measures of the iTUG’s responsiveness were reported by Picardi et al. [31] showing a small to medium improvement after the intervention. The correlation between iTUG and MiniBESTest was not significant [31].

### Critical appraisal of included studies

Detailed results of the methodological quality assessment are shown in Supplement 4. In several studies, information on the iTUG measurement procedures were missing and needed to be requested: performance of practice trials before the iTUG measurement, use of walking aids, attachment of the sensors (i.e., body-fixed to the skin vs. body-worn over clothing), performance instructions (e.g., preferred speed or as fast as possible), measurement time points (e.g., how many days/weeks after the intervention) and analysis of the repeated measurements (e.g., mean, best-of all iTUG trials). Ninety-three percent of the authors contacted provided the missing information.

## Discussion

This scoping review presents an overview on intervention studies applying iTUG measurements in groups of older people. Patients with idiopathic normal pressure hydrocephalus (PwiNPH), patients with Parkinson's disease (PwPD), patients with elective joint replacement and older people participating in exercise and rehabilitation interventions were identified as clinical use cases. The following discussion focuses on clinical iTUG applications that are ready for use or at least approaching this state. We focus on results that are not only statistically significant and detectable in terms of a minimal detectable change (MDC) but which are also relevant from a clinical perspective classfied by the concept of minimal important difference (MID). There is inconsistency in the literature regarding the terms MID, MCID, MIC, MCIC. For the purpose of this scoping review, the currently most widely acclaimed terminology MID [42] will be used reflecting the smallest difference in the TUG score that clinicians and patients perceive as relevant or meaningful and that thereby may contribute to decision making in therapy planning [43, 44].

### Clinical use case 1: stratification, evaluation and monitoring of PwiNPH

The most mature use case was identified in PwiNPH. The diagnosis of iNPH is based on imaging, medical history and a positive tap-test. The response of a tap-test is defined as a ≥ 1 point improvement on the iNPH grading scale assessing the severity of gait disturbance, cognitive impairment and urinary incontinence [24]. The included studies used the iTUG to quantitatively assess gait before and after a tap-test. Three studies observed an iTUG improvement ranging from 2 to 8.7 s after the tap-test. MID/MDC TUG values in PwiNPH were reported to be 3.6 s [45] to 5 s [22].

The change in mobility performance are key to recommend and perform a VPS surgery. In case of a positive tap-test result, a VPS is recommended. The results of the included studies show a reduction of the TUG total duration in the tap-test-positive groups after tap-test and after a subsequent VPS surgery confirming the diagnosis. This indicates that the iTUG could serve as a digital mobility outcome to stratify patients regarding eligibility for subsequent VPS surgery and to evaluate surgery success. The iTUG could also be useful for monitoring patients to control the VPS function [22], and potentially adapt valve function if needed.

In PwiNPH the iTUG is used on a n = 1 basis, with each patient serving as his or her own control. This offers the possibility of individualised clinical management. The recommendation of the most recent guideline from Japan for an invasive treatment (i.e., VPS surgery) is based on MRI imaging, medical history and results of the iTUG. The iTUG can be performed objectively as well as repeated and reproduced if needed [46].

The results of the included studies do not show to what extent specific segments of the iTUG could be particularly relevant for this use case.

### Clinical use case 2: stratification, evaluation and monitoring of PwPD

A common clinical challenge is the differentiation between neurodegenerative PD and vascular parkinsonism. Cerebrovascular lesions can cause manifestations similar to PD. However, most of these patients are older at onset and have more prominent symptoms of the lower extremities [47]. l-dopa therapy is often started without objective tests, although this treatment is not indicated in patients with mixed pathology and patients may experience side effects such as orthostatic symptoms or nausea [47]. In this review, two studies used the iTUG to measure mobility in OFF and ON stages of medication with each patient serving as his or her own control [25, 26]. The MDC is around 3.5 s [44]. A change of > 3.5 s was found in one of the included PwPD studies [25]. In the other study, the iTUG difference was smaller and may not have been subjectively noticeable because the participants did not show any physical capacity deficits at baseline, as shown by mean iTUG values of < 9 s. In this clinical use case the iTUG could be useful for stratification purposes, e.g., de-prescribing medication for patients with parkinsonism due to vascular disease or patients with atypical PD.

PwPD are amongst the most analyzed participants in studies using iTUG approaches [13–15], therefore it is not surprising that this patient group was identified as a clinical use case for the iTUG. However, mobility problems, e.g., small shuffling steps, usually worsen with disease severity and are not regarded as pre-clinical or early stage symptoms of PD [47]. Therefore, the iTUG has a potential for evaluating and monitoring of PwPD with moderate-to-severe stages of PD [13] (Hoehn and Yahr stages II, III and IV) but it may not be suitable to establish the diagnosis of PD.

The iTUG also allows the specific analysis of turning maneuvers in PwPD, which is of particular relevance in this population, especially when the disease progresses [48]. In the included studies, changes in the turn segments were more likely to show significant improvements with therapy than the other segments [25, 26, 30]. The total iTUG duration and turning segment durations should be compared with a clinical gold standard and with Patient-Reported Outcome Measures (PROMs) such as the MDS-UPDRS-II/III. The iTUG may therefore be used as secondary endpoint. Previous research showed excellent reliability of wearable inertial sensor-based iTUG measurements in PwPD [49] as well as feasibility and sensitivity to detect mobility deficits in patients with PD at home [50].

Another perspective on iTUG application is its use to analyse freezing symptoms of PwPD. One study that classified PwPD by their history of freezing of gait found that turn segments were longer in freezers who showed greater improvements with medication [25]. Consequently, observing changes in the turning segment of the iTUG might be a relevant criterion in addition to the total duration for monitoring clinically relevant changes in PwPD. However, for the analysis of freezing of gait, multisensory approaches would be necessary (e.g., additional sensors worn on shoes [21, 22]), which are more complex, cost-intensive, and thus have a more limited clinical applicability than unisensory approaches. Ideally, the iTUG assessment of PwPD should have a mandatory seven-day mobility measurement, as this approach will also capture fluctuating and rare events, e.g., freezing or falls [51].

### Clinical use case 3: monitoring after joint replacement surgery

Another potential use case is the use of the iTUG to evaluate surgical procedures, e.g., by monitoring patients before and after elective joint replacement surgery. Typically, the mobility of patients deteriorates in the first 2–3 weeks after the operation before significant improvements in physical capacity are observed. This is also shown in the results of two studies [27, 28]. MDC scores for iTUG measurements for knee (2.3 s [52]) and hip arthroplasty (1.6 s [53]) are reported in studies following patients for 6 months. Participants in both studies showed statistically significant and detectable iTUG changes. Participants with low mobility impairment before knee surgery had greater improvements in iTUG total duration 12 weeks after surgery (4.9 s) compared to those with moderate mobility impairment preoperatively (0.9 s) [27].

The iTUG could qualify as a clinical endpoint while changes in physical activity, pain, and quality of life may serve as additional endpoints for these patient groups. The iTUG also has the potential to compare the results of home-based and inpatient rehabilitation [54] as the assessment could be performed in an unsupervised manner. The available data on the individual TUG segments in the included studies do not allow any additional conclusions.

### Clinical use case 4: evaluation of exercise and rehabilitation interventions

Six out of ten studies who used the iTUG to evaluate exercise and rehabilitation interventions showed a statistically significant improvement of the total iTUG duration [29–33, 36] ranging from 0.5 to 3.6 s.

In the three studies with PwPD moderate to large effects were reported from participants of LSVT-BIG®, Mat Pilates and inpatient rehabilitation (physiotherapy/occupational therapy) ranging from 1.6 to 1.9 s. These results are in line with results of other studies reporting positive effects of LSVT-BIG® and physiotherapy on motor performance [55, 56]. In the inpatient rehabilitation study, iTUG measures were responsive to the interventions applied. It remains unclear whether the differences in iTUG total duration are perceived as meaningful, as to the best of our knowledge no MID is available for exercise or rehabilitation interventions in PwPD. Particularly in the two studies with short baseline iTUG times < 10 s [29, 30], more challenging assessments such as the Community Balance and Mobility Scale (CMB) [57]) might be more appropriate to identify relevant changes in physical capacity.

Outpatient programs showed statistically significant changes being 0.5 s for the “Better Bones” strength and balance training and 2.1 s for the back school rehabilitation. The MDC reported in the “Better Bones” study was 0.8 s [32]. A MID value of 0.8 to 1.4 was reported for the TUG in a study observing outpatients with hip osteoarthritis undergoing exercise therapy [58]. Although they constitute a diffent patient population it can be assumed that the back school participants experienced an MID in their iTUG performance. This is also supported by the fact that the participants in this study surpassed the normal vs. below normal mobility threshold of 12 s [41]. It seems unlikely that the 0.5 s iTUG change in the “Better Bones” study was perceived as a meaningful change, particularly as the participants started with fast iTUG baseline durations of 6.3 s. It is noticeable that even if the total duration did not change significantly after the stepping intervention, the time needed for turn 2 was significantly shorter.

Inpatient rehabilitation (physiotherapy/occupational therapy) participants with peripheral neuropathy showed statistically significant changes of 3.6 s in iTUG performance at the end of rehabilitation. Day care and care home exercise programs for older people > 80 years did not show statistically significant improvements regarding iTUG performance although numerically large differences from + 3.5 s (slower iTUG) to − 4.9 s (faster iTUG) were reported. Standard deviations were large in all of the three inpatient studies indicating a sample size problem. In the day care study [38], sit-to-stand and walking segments improved, but the total iTUG duration did not change significantly.

Since only one study examined the responsiveness of the iTUG [31], it remains largely unclear whether the interventions in the other exercise and rehabilitation studies were actually ineffective or whether the applied iTUG procedure was not able to detect a true change.

In the two studies applying assistive devices significance was not reported. Therefore a use case could not be identified.

### Methodological limitations

Most of the included studies were pilot studies. Their relevance is therefore limited due to their small sample size. Because of insufficient reporting on iTUG methodology in the included studies, we see a urgent need for a consensus to standardize the iTUG measurement and its reporting. This would improve the interpretability of results. In Textbox 1, we suggest recommendations for minimum reporting in studies using iTUG approches.

Textbox 1: Recommendations for minimum reporting in studies using iTUG approches

- iTUG technology and device
- Walking distance
- Site of attachment
- Fixation of sensors and devices
- Exact wording of the verbal instructions
- (Non-) performance of practice trials
- Number of repetitions

### Recommendations and future directions

The iTUG has a high potential to be implemented by clinicians. iTUG algorithms enable a standardized and reproducible measurement of the TUG. An instrumented measurement allows the assessor to concentrate on observing and safeguard the patient without being distracted, e.g. when handling a stopwatch. The use of smartphones extends the scope of applicability from analogue inpatient measurement to remote monitoring and self-assessment in outpatient and inhome settings. The latter will be needed for an inclusive medical approach when access to inpatient services will be increasingly limited.

The need for further validation studies adhering to the COSMIN terminology of measurement properties is once again emphasized by the WHO Locomotor Capacity Working Group [10].

A recent systematic review [59] of MIDs for different balance measures used with older people in research and clinical settings shows that MIDs are still pending for many health conditions. Future studies should aim to determine iTUG responsiveness and MID thereby highlighting the ability of the iTUG to detect clinically important change [10, 60].

In some use cases other or more challenging assessments (e.g., CBM, MiniBEST test) should be used as additional measures to perform a comprehensive assessment of physical capacity. This is particularly relevant for patients with a higher performance level where the iTUG might have ceiling effects. Multiple repetitions of the iTUG (e.g., three to five) during one assessment could be beneficial to eliminate the variance in performance between the runs [12].

For the time being, we consider the analysis of kinematic parameters as clinically exploratory. It is unlikely that clinical experts will be familiar with angular velocities and acceleration values in the foreseeable future.

From a global perspective, feasible and widely applicable assessments are required, especially for use in low- and middle-income countries [10]. The iTUG holds great potential in this regard, as the measurement can be carried out almost at any time and in any location where a chair, a 3 m space and, e.g., a smartphone is available. Data gathered by using the iTUG could be digitally and automatically stored, shared and analyzed over long distances, enabling continuous monitoring and, if indicated, rapid action planning.

## Conclusion

This scoping review reveals first use cases for the application of an iTUG for PwiNPH and PwPD. The iTUG could also be used to monitor and evaluate joint replacement surgery as well as exercise and rehabilitation interventions. Methodological and reporting limitations of the included studies currently affect interpretability. Therefore a consensus is required to guarantee a more harmonized performance and reporting in future studies and in clinical practice.

### Supplementary Information

Below is the link to the electronic supplementary material.

Supplementary file1 (PDF 97 KB)

Supplementary file2 (PDF 55 KB)

Supplementary file3 (PDF 51 KB)

Supplementary file4 (PDF 148 KB)

Supplementary file5 (PDF 57 KB)

Supplementary file6 (PDF 264 KB)

## Data Availability

No datasets were generated or analysed during the current study.
