# Supplementary material for: “TiC-TUG”: technology in clinical practice using the instrumented timed up and go test—a scoping review
Source: Aging Clin Exp Res. 2024 Apr 27;36(1):100. doi: 10.1007/s40520-024-02733-7 (PMC11055724; doi:10.1007/s40520-024-02733-7)
Supplement: Supplementary file 2 — Supplementary file2 (PDF 55 KB) [file 40520_2024_2733_MOESM2_ESM.pdf]

Supplement 2: Inclusion and exclusion criteria

Criterion	Include	Exclude
Publication year	2012-2022	Studies published before 2012
Publication type	Peer-reviewed articles, full-text available	Posters, short communications, conference papers, dissertations, reviews
Sample size	*N* ≥ 20 available for analysis	*N* < 20 available for analysis
Mean age	Mean age ≥ 60 years	Mean age < 60 years
Population	Healthy older adults, common age-associated diseases (e.g., Parkinson, Arthrosis)	Early acquired or congenital diseases, typically not associated with aging (e.g., MS); orphan diseases: less than five in 10,000 persons in Europe, as defined by the European Regulation on orphan medicinal products, (e.g., Huntington)
Study design	Prospective studies, at least 2 measurement time points (e.g., follow up, post-surgery, Parkinson on/off state)	Cross-sectional studies
Study aim	Studies using iTUG to measure pre-post changes following interventions with older adults (e.g., medication, training, surgery, tap-test, assistive device)	Studies using the iTUG to predict clinical outcomes
Type of data	Primary and secondary analyses	
iTUG walking distance	3 meters	Other distances, modified walking distances (e.g., door passage, turning in front of wall)
iTUG technology	Technology body-worn during TUG (e.g., wearable sensors, smartphone)	Video-based measurement, instrumented walkway, laser
iTUG procedure	Technology measures during the test	Stopwatch measurement with subsequent data entry
Reported data	Minimum requirement: reported duration of total iTUG or iTUG segments (e.g., turning, sit-to-stand)	No iTUG total/segment duration data reported
