# Supplementary material for: “TiC-TUG”: technology in clinical practice using the instrumented timed up and go test—a scoping review
Source: Aging Clin Exp Res. 2024 Apr 27;36(1):100. doi: 10.1007/s40520-024-02733-7 (PMC11055724; doi:10.1007/s40520-024-02733-7)
Supplement: Supplementary file 3 — Supplementary file3 (PDF 51 KB) [file 40520_2024_2733_MOESM3_ESM.pdf]

**Supplement 3:Search string for PubMed/MEDLINE**

PubMed: [tiab], [ti], [tw], [mh], [mh:noexp], [sh]
Ovid: Exp "…"/, (… OR …).ti,ab,kf., (…) ADJ3 (…)

| | |
|---|---|
| (<br>TUG[tiab] OR<br>iTUG[tiab] OR<br>"Timed Up and Go"[tiab] OR<br>"Timed Up Go"[tiab]<br>)<br>AND | A1 |
| (<br>Acceleromet*[**tw**] OR<br>instrumented[tiab] OR<br>sensor*[tiab] OR<br>"inertial measurement unit*"[tiab] OR<br>IMU[tiab] OR<br>Gyroscope*[tiab] OR<br>Magnetometer*[tiab] OR<br>"mobile sensing"[tiab] OR<br><br>"Wearable Electronic Devices"[**Mesh:noexp**] OR<br>device*[tiab] OR<br>electronic*[tiab] OR<br><br>"Cell Phone*"[**tw**] OR<br>Smartphone*[**tw**] OR<br>"Smart phone*"[tiab] OR<br>"Mobile device*"[tiab] OR<br>"Mobile phone*"[tiab] OR<br>Tablet*[tiab] OR<br><br>"Mobile App*"[**tw**] OR<br>app-based[tiab] OR<br><br>"mobile health"[tiab] OR<br>mHealth[tiab] OR<br>ehealth[tiab] OR<br>software[tiab] OR<br>digital[tiab] OR<br>technolog*[tiab]<br>) | A2<br><br>Technology[Mesh]?<br>Electronics[Mesh]?<br><br>"Wearable Electronic Devices" ><br>Fitness Trackers, Hearing Aids,<br>Smart Glasses<br><br>[tw] inkl. Meshterms |
| AND<br>(<br>2012:2022[dp]<br>) | Filter<br><br>Ab 2012 |
| NOT<br>(<br>(infant[mh] OR child[mh] OR adolescent[mh] OR "Young Adult"[mh]) **NOT** (aged[mh] OR "Middle Aged"[mh])<br>) | Filter<br><br>Erwachsene > 60<br><br>(rund 40 Treffer weniger) |
