# Supplementary material for: “TiC-TUG”: technology in clinical practice using the instrumented timed up and go test—a scoping review
Source: Aging Clin Exp Res. 2024 Apr 27;36(1):100. doi: 10.1007/s40520-024-02733-7 (PMC11055724; doi:10.1007/s40520-024-02733-7)
Supplement: Supplementary file 4 — Supplementary file4 (PDF 148 KB) [file 40520_2024_2733_MOESM4_ESM.pdf]

Supplement 4: Quality Assessment Tool for Before-After (Pre-Post) Studies with No Control Group

**First author (Year)**	1. objective clearly stated	2. eligibility criteria clearly described	3. participants representative of population	4. all eligible participants enrolled	5. sample size sufficiently large	6. intervention clearly described	7. outcome measures clearly defined	8. assessors blinded to participants' interventions	9. loss to follow up 20% or less	10. statistical examination of pre-post changes	11. ≥ 2 iTUG repetitions per assessment timepoint	12. multilevel structure
**Patients with idiopathic normal pressure hydrocephalus (PwiNPH) – surgical and invasive procedures**												
Ferrari (2020)	yes	yes	yes	yes	NR	yes	no	NR	NR	no	yes	NA
Ferrari (2022)	yes	yes	yes	yes	yes	yes	no	NR	yes	yes*	yes	NA
Yamada (2019)	yes	yes	yes	yes	NR	yes	yes	NR	yes	no	yes	NA
Ishikawa (2019)	yes	no	yes	no	NR	yes	yes	NR	yes	yes	yes	NA
**Patients with Parkinson's disease (PwPD) - pharmacological intervention**												
Dibilio (2017)	yes	yes	yes	NR	NR	yes	no	NR	yes	yes	yes	NA
Miller Koop (2018)	yes	yes	yes	NR	NR	yes	no	yes	NR	yes	yes	NA
**Orthopedic conditions – elective surgery**												
Bloomfield (2019)	yes	yes	yes	NR	NR	yes	no	NR	no	yes	yes	NA
Perelgut (2020)	yes	yes	yes	NR	yes	yes	no	NR	yes	yes	yes	NA
**Exercise and rehabilitation interventions in different settings and populations**												
**Patients with Parkinson's disease (PwPD)**												
Flood (2020)	yes	yes	yes	NR	NR	yes	no	NR	NR	yes	yes	NA
Mollinedo-Cardalda (2018)	yes	yes	yes	yes	yes	yes	no	no	yes	yes	NR	NA
Picardi (2020)	yes	yes	yes	NR	NR	no	yes	NR	NR	yes	yes	NA
**Participants recruited in outpatient settings**												
Smith (2021)	yes	yes	yes	NR	yes	yes	no	NR	no	yes*	NR	NA

**First author (Year)**	1. objective clearly stated	2. eligibility criteria clearly described	3. partici-pants re-presenta-tive of po-pulation	4. all eligi-ble partici-pants en-rolled	5. sample size suffi-ciently large	6. interven-tion clearly described	7. outcome measures clearly de-fined	8. asses-sors blinded to partici-pants' in-terventions	9. loss to follow up 20% or less	10. statisti-cal exami-nation of pre-post changes	11. ≥ 2 iTUG repe-titions per assess-ment timepoint	12. multi-level struc-ture
Celletti (2020)	yes	yes	yes	NR	NR	yes	no	NR	yes	yes	NR	NA
Doheny (2013)	yes	no	yes	NR	NR	yes	no	NR	yes	yes	yes	NA
Williams (2021)	yes	yes	yes	yes	no	yes	no	yes	no	yes	no	NA
**Participants recruited in inpatient and institutionalized settings**												
Caronni (2019)	yes	yes	yes	NR	NR	yes	yes	NR	yes	yes	yes	NA
Cancela Carral (2017)	yes	yes	yes	NR	NR	yes	no	no	no	yes	NR	NA
Cancela Carral (2019)	yes	yes	yes	NR	no	yes	no	NR	yes	yes	NR	NA
**Assistive devices**												
Toosizadeh (2020)	yes	yes	yes	NR	NR	yes	no	NR	NR	no	yes	NA
Yalla (2014)	yes	yes	yes	NR	yes	yes	no	NR	NR	yes*	NR	NA

Notes: yes* = if intention-to-treat analysis was reported

Items and specific criteria for quality rating

Items	yes	no
1. Was the study question or objective clearly stated?	if study question/objective clearly stated	if study question/objective not clearly stated
2. Were eligibility/selection criteria for the study population prespecified and clearly described?	if criteria prespecified and clearly described	if criteria not prespecified/not sufficiently described
3. Were the participants in the study representative of those who would be eligible for the test/service/intervention in the general or clinical population of interest?	yes for all studies, because inclusion criteria were age > 60 years; congenital, non-aged-related, or orphan diseases were excluded	
4. Were all eligible participants that met the prespecified entry criteria enrolled?	if flowchart or description of enrollment provided	if further in/exclusion criteria were added after recrtuiting or enrollment with no reason
5. Was the sample size sufficiently large to provide confidence in the findings?	if sample size calculation reported and sufficient sample size achieved	if sufficient sample size was not achieved
6. Was the test/service/intervention clearly described and delivered consistently across the study population?	if intervention is clearly described	if information on duration, frequency, type of intervention missing
7. Were the outcome measures prespecified, clearly defined, valid, reliable, and assessed consistently across all study participants?	if outcome measures prespecified ad cleary described	if information on iTUG distance, repetitions, sensors, body placement, instruction missing (see table 2)
8. Were the people assessing the outcomes blinded to the participants' exposures/interventions?	if assessors were blinded	if assessors were not blinded
9. Was the loss to follow-up after baseline 20% or less? Were those lost to follow-up accounted for in the analysis?	yes: if loss to follow-up ≤ 20%;<br>yes*: if intention-to-treat analysis was reported	if loss to follow-up >20%
10. Did the statistical methods examine changes in outcome measures from before to after the intervention? Were statistical tests done that provided p values for the pre-to-post changes?	yes: if p values reported for pre-post changes;<br>yes*: if confidence intervals are also available for all reported data	if descriptive statistics only if no p value reported
11. Were outcome measures of interest taken multiple times before the intervention and multiple times after the intervention (i.e., did they use an interrupted time-series design)?	if > 1 iTUG repetition per measurement timepoint	if only 1 iTUG repetition
12. If the intervention was conducted at a group level (e.g., a whole hospital, a community, etc.) did the statistical analysis take into account the use of individual-level data to determine effects at the group level?	NA for all studies, none of the included studies was conducted at a group level	
