# Supplementary figures and images for: “TiC-TUG”: technology in clinical practice using the instrumented timed up and go test—a scoping review
Source: Aging Clin Exp Res. 2024 Apr 27;36(1):100. doi: 10.1007/s40520-024-02733-7 (PMC11055724; doi:10.1007/s40520-024-02733-7)

## Supplement 5: PRISMA-ScR flow diagram

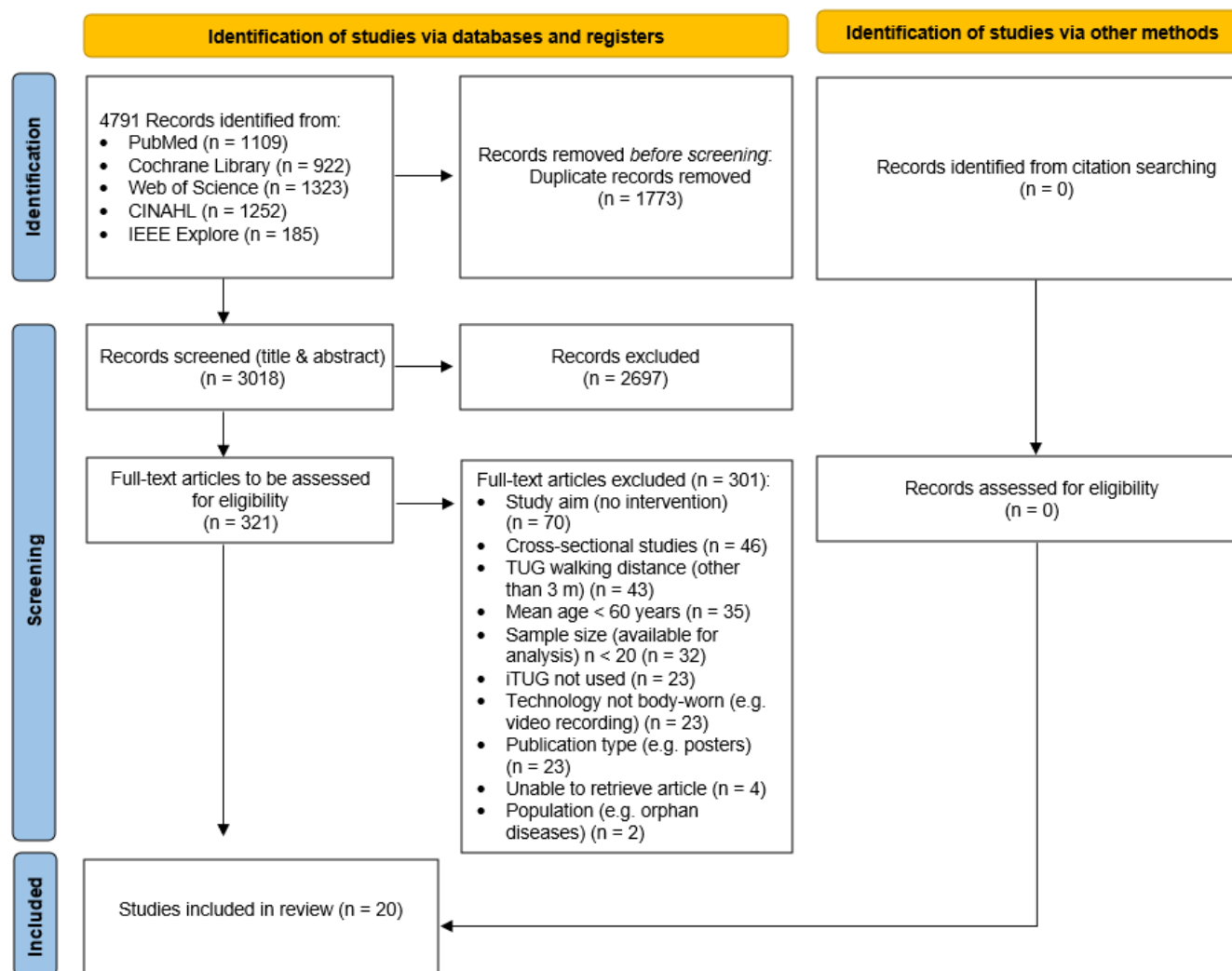

Supplement: Supplementary file 5 — Supplementary file5 (PDF 57 KB) [file 40520_2024_2733_MOESM5_ESM.pdf]
