# Supplementary material for: “TiC-TUG”: technology in clinical practice using the instrumented timed up and go test—a scoping review
Source: Aging Clin Exp Res. 2024 Apr 27;36(1):100. doi: 10.1007/s40520-024-02733-7 (PMC11055724; doi:10.1007/s40520-024-02733-7)
Supplement: Supplementary file 6 — Supplementary file6 (PDF 264 KB) [file 40520_2024_2733_MOESM6_ESM.pdf]

Supplement 6: Pre-Post differences within groups reported for iTUG total duration and segments including “trends” (non-significant changes)

	Groups	Pre-post conditions/timepoints	iTUG total	Sit to stand	Walk 1	Turn 1	Walk 2	Turn 2	Stand to sit	Notes
**Patients with idiopathic normal pressure hydrocephalus (PwiNPH) – surgical and invasive procedures**										
Ferrari (2022)	IG	before tap-test vs. 6 months after ventriculoperitoneal shunt surgery (VPS)	+**	+**	total walk: +**	N/A			+**	
Ishikawa (2019)	IG	baseline vs. day after tap-test	N/A	+	+	+	+	o	+	*CG: post-intervention data N/A*
		baseline vs. 1w after VPS		+	+*	+	+*	+*	+*	
		tap-test vs. VPS		o	+*	+	+	+	+	
**Patients with Parkinson’s disease (PwPD) - pharmacological intervention**										
Dibilio (2017)	IG	off vs. on medication	+*	+	+*	+**	+*	+**	+	
Miller Koop (2018)	IG	off vs. on medication	+*	+	total walk: +	+*	N/A	turn to sit: -		
**Orthopedic conditions – elective surgery**										
Bloomfield (2019)	moderate function	baseline vs. 2w after TKR	-**	(-)	(-)	(-)	(-)	N/A	(-)	*data was extracted from bar chart, no values or significances reported*
		baseline vs. 6w after TKR	-	(o)	(+)	(+)	(-)		(o)	
		baseline vs. 12w after TKR	+	(-)	(+)	(+)	(+)		(o)	
	low function	baseline vs. 2w after TKR	-	(o)	(-)	(-)	(-)		(-)	
		baseline vs. 6w after TKR	+	(+)	(+)	(+)	(+)		(o)	
		baseline vs. 12w after TKR	+**	(+)	(+)	(+)	(+)		(+)	
**Exercise and rehabilitation interventions in different settings and populations**										
**Patients with Parkinson’s disease (PwPD)**										
Mollinedo Cardalda (2018)	IG (mat pilates)	baseline vs. end of intervention	+**	+	+*	+	+*	+*	N/A	
		end of intervention vs. 4w after	+	-*	-	+	-*	+*		
	CG (calisthenics)	baseline vs. end of intervention	-	-	-	+	-	-		
		end of intervention vs. 4w after	-	-	-	+	-	-*		

<table>
<tr><th></th><th>Groups</th><th>Pre-post conditions/timepoints</th><th>iTUG total</th><th>Sit to stand</th><th>Walk 1</th><th>Turn 1</th><th>Walk 2</th><th>Turn 2</th><th>Stand to sit</th><th>Notes</th></tr>
<tr><td colspan="11">Participants recruited in outpatient settings</td></tr>
<tr><td rowspan="2">Smith (2021)</td><td rowspan="2">IG
(„Better Bones“)</td><td rowspan="2">baseline vs. after intervention</td><td rowspan="2">+**</td><td rowspan="2">+**</td><td>+**</td><td rowspan="2">+*</td><td rowspan="2" colspan="2">N/A</td><td rowspan="2">+</td><td></td></tr>
<tr><td>total walk: +*</td><td></td></tr>
<tr><td>Celletti (2020)</td><td>IG</td><td>baseline vs. after back school treatment</td><td>+**</td><td>+*</td><td colspan="4">N/A</td><td>+</td><td>measurement timepoint not specified</td></tr>
<tr><td rowspan="4">Doheny (2013)</td><td rowspan="4">IG
(step exercise)</td><td rowspan="2">baseline (T1) vs. 4w after T1 (T2)</td><td rowspan="2">+</td><td rowspan="2">+</td><td colspan="3">N/A</td><td>+*</td><td>o</td><td rowspan="4">two baseline measures: T1 = first baseline, T2 = 4w after baseline before start of intervention</td></tr>
<tr><td colspan="5">walk to sit: +</td></tr>
<tr><td rowspan="2">baseline (T1) vs. 4w after end of interv. (T3)</td><td rowspan="2">+</td><td rowspan="2">+</td><td colspan="3">N/A</td><td>+*</td><td>-</td></tr>
<tr><td colspan="5">walk to sit: +</td></tr>
<tr><td rowspan="2">Williams (2021)</td><td>IG
(Tai Chi)</td><td rowspan="2">baseline vs. 6 months after baseline</td><td rowspan="2">N/A</td><td>+</td><td>+</td><td>-</td><td>+</td><td rowspan="2">N/A</td><td rowspan="2">N/A</td><td></td></tr>
<tr><td>CG
(usual care)</td><td>+</td><td>-</td><td>-</td><td>-</td><td></td></tr>
<tr><td colspan="11">Participants recruited in inpatient and institutionalized settings</td></tr>
<tr><td>Caronni (2019)</td><td>IG</td><td>baseline vs. end of physiotherapy/occupational therapy rehabilitation program</td><td>+**</td><td>+**</td><td>+**</td><td>+**</td><td>+**</td><td colspan="2">turn and sit: +*</td><td></td></tr>
<tr><td rowspan="3">Cancela Carral (2017)</td><td>IG_A (aerobic)</td><td rowspan="3">baseline vs. 1w after end of intervention</td><td>+</td><td>+</td><td>+*</td><td>+</td><td>+*</td><td rowspan="3">N/A</td><td>+</td><td></td></tr>
<tr><td>IG_B (resistance)</td><td>+</td><td>+*</td><td>+*</td><td>+</td><td>+*</td><td>+</td><td></td></tr>
<tr><td>IG_C (mobility)</td><td>-</td><td>-*</td><td>-</td><td>-*</td><td>+</td><td>-*</td><td></td></tr>
<tr><td rowspan="2">Cancela Carral (2019)</td><td>IG
(strength training)</td><td rowspan="2">baseline vs. last week of intervention</td><td>+</td><td>-</td><td>+</td><td>-*</td><td>+</td><td rowspan="2">N/A</td><td>-</td><td></td></tr>
<tr><td>CG</td><td>-*</td><td>-*</td><td>-</td><td>-</td><td>+</td><td>-*</td><td></td></tr>
<tr><td colspan="11">Assistive devices</td></tr>
</table>

<table>
<tr><th></th><th>Groups</th><th>Pre-post conditions/timepoints</th><th>iTUG total</th><th>Sit to stand</th><th>Walk 1</th><th>Turn 1</th><th>Walk 2</th><th>Turn 2</th><th>Stand to sit</th><th>Notes</th></tr>
<tr><td rowspan="4">Toosizadeh (2020)</td><td rowspan="2">moderate function</td><td>no calf vibration vs. 30Hz</td><td>(-)</td><td>(-)</td><td>(+)</td><td>-</td><td>(+)</td><td colspan="2">-</td><td></td></tr>
<tr><td>no calf vibration vs. 40Hz</td><td>(-)</td><td>(-)</td><td>(+)</td><td>-</td><td>(+)</td><td colspan="2">-</td><td></td></tr>
<tr><td rowspan="2">low function</td><td>no calf vibration vs. 30Hz</td><td>(+)</td><td>(-)</td><td>(-)</td><td>+</td><td>(-)</td><td colspan="2">+*</td><td></td></tr>
<tr><td>no calf vibration vs. 40Hz</td><td>(+)</td><td>(-)</td><td>(-)</td><td>+*</td><td>(-)</td><td colspan="2">+</td><td></td></tr>
</table>

Notes: articles that do not provide data on iTUG segments are not displayed in this table, + = improvement, - = worsening, o = no difference, *p < .05, ** p < .01 compared to baseline, CG = control group, IG = intervention group, N/A = not available, () = significance not reported, TKR = total knee replacement, VPS = ventriculoperitoneal shunt surgery
